# Der „schwierige“ Patient – Vestibularisdiagnostik unter erschwerten Bedingungen: Teil 1: Anamnese und klinisch-neurootologische Untersuchung

**DOI:** 10.1007/s00106-022-01179-5

**Published:** 2022-05-18

**Authors:** Julia Dlugaiczyk

**Affiliations:** Klinik für Ohren‑, Nasen‑, Hals- und Gesichtschirurgie & Interdisziplinäres Zentrum für Schwindel und neurologische Sehstörungen, Universitätsspital Zürich (USZ), Universität Zürich (UZH), Frauenklinikstrasse 24, 8091 Zürich, Schweiz

**Keywords:** Anamnese, Schwindel, Vestibulookulärer Reflex, SO STONED, DISCOHAT, History taking, Vertigo, Vestibulo-ocular reflex, SO STONED, DISCOHAT

## Abstract

Der Patient mit dem Leitsymptom Schwindel stellt häufig eine Herausforderung für den Hals-Nasen-Ohren-Arzt dar. Die folgende Artikelserie beleuchtet unterschiedliche Aspekte des „schwierigen“ Schwindelpatienten. Der vorliegende erste Teil widmet sich den Besonderheiten und Fallstricken bei der Anamneseerhebung und der klinisch-neurootologischen Untersuchung. Dabei werden situationsspezifische Lösungsansätze zu folgenden Themen der Anamneseerhebung aufgezeigt: Definition von Erwartungen und Zielen, „ausschweifende“ Anamnese, Beschreibung des Symptoms Schwindel, mehrere Schwindelentitäten bei einem Patienten, Diskrepanz zwischen Symptomschwere und vestibulären Befunden, kognitive Verzerrungen und der Umgang mit Emotionen. Des Weiteren werden praxisbezogene Hinweise für die neurootologische Untersuchung von Patienten mit Halswirbelsäulenproblemen und Augenbewegungsstörungen sowie bei ängstlichen Patienten gegeben.

## Abstract (en)

Patients presenting with vertigo or dizziness may pose a real challenge for the clinical otorhinolaryngologist. This series of articles covers different aspects of the “difficult” dizzy patient. The first part is dedicated to pearls and pitfalls in history taking and clinical neurotological examination. It suggests possible solutions for challenging situations in history taking, such as definition of the expectations and aims, patients presenting with a long-winded history, patients’ description of the symptom “vertigo”, multiple vestibular syndromes in one patient, discrepancy between subjective symptoms and objective vestibular findings, cognitive bias and dealing with emotions. Furthermore, it offers practically oriented tips for the neurotological examination of patients with problems of the cervical spine, oculomotor disorders and anxiety.

## Lernziele

Nach Absolvieren dieser Fortbildungseinheit …

- können Sie bei Patienten mit dem Leitsymptom Schwindel eine strukturierte und umfassende Anamnese in 15 min durchführen.
- erfassen Sie die verschiedenen Dimensionen von Schwindelbeschwerden mit den Merkhilfen SO STONED und DISCOHAT.
- erkennen Sie Hinweise auf funktionelle Schwindelbeschwerden in Anamnese und klinischer Untersuchung.
- können Sie Techniken zur neurootologischen Untersuchung ängstlicher Patienten anwenden.

### Fallbeispiel

Als die 30-jährige Patientin im Wartezimmer aufgerufen wird, erhebt sie sich langsam vom Stuhl, greift suchend nach dem Arm ihrer Mutter und kommt mit langsamen Schritten auf die behandelnde Ärztin zu. Auf dem gemeinsamen Weg ins Untersuchungszimmer bewegt sie sich vorsichtig wie auf Glatteis, stets bemüht, den Kopf gerade zu halten. Dort angekommen, legt sie eine dicke Mappe mit Vorbefunden auf den Tisch. Auf Nachfrage berichtet sie, dass alles mit einem Lagerungsschwindel vor etwa 2 Jahren begonnen habe. Dieser sei erfolgreich reponiert worden, unmittelbar danach habe sie einen Druck auf Höhe des Atlaswirbels bemerkt. Seither leide sie unter mehreren Formen von Schwindel, Tinnitus und „panzerartigen Schmerzen“ im Bereich der Halswirbelsäule (HWS) und des Brustkorbs. Die Beschwerden seien über die Zeit progredient, seit 3 Monaten könne sie deswegen nicht mehr das Haus verlassen. Als Beifahrerin im Auto trage sie eine Halskrause aus Angst vor Erschütterungen der HWS. Während des gesamten Gesprächs hält die Patientin den Kopf komplett ruhig. Auf dem Weg zur Untersuchungsliege – mit Festhalten am Arm der Ärztin – sagt sie: „Bewegen Sie bei der Untersuchung bloß nicht meinen Kopf!“

## Hinführung zum Thema

Bei der Patientin aus der Kasuistik handelt es sich um keinen konstruierten Fall, sondern um eine ganz reale Patientin aus unserer Schwindelsprechstunde. Beim Lesen hatte sicher jeder von Ihnen „schwindelerregende“ Erinnerungen an eigene Patienten mit einer ähnlichen Geschichte. Die folgende Artikelserie soll Ihnen auf Basis dieser Kasuistik eine Hilfestellung im Umgang mit „schwierigen“ Schwindelpatienten bieten. Sie zeigt Ihnen verschiedene situationsbezogene Lösungsansätze auf und geht dabei über eine bloße Sammlung von „Tipps und Tricks“ hinaus – dies würde der Komplexität der vestibulären Krankheitsbilder und den betroffenen Patienten nicht gerecht werden. Der Autorin ist dabei bewusst, dass wir alle in unserem klinischen Alltag mit begrenzten Ressourcen konfrontiert sind (Zeit, Untersuchungsmethoden, Personal) und daher nicht alle hier präsentierten Lösungsansätze universell umsetzbar sind. Wenn Sie eine oder zwei Ideen in Ihren klinischen Alltag integrieren können, ist Ihnen und Ihren Patienten sicherlich bereits geholfen.

Der vorliegende erste Teil der Serie widmet sich den Besonderheiten und Fallstricken bei der Anamneseerhebung und der klinisch-neurootologischen Untersuchung. In den nachfolgenden Artikeln werden schwierige Aspekte der apparativen **Vestibularisdiagnostik**

Vestibularisdiagnostik,

der Umgang mit besonderen Patientengruppen (z. B. Kinder, geriatrische Patienten, Patienten mit kognitiven Beeinträchtigungen), differenzialdiagnostische „Knacknüsse“ und schwierige Situationen der **Patient-Arzt-Interaktion**

Patient-Arzt-Interaktion

näher beleuchtet. Dabei steht der typische Patient einer Schwindelsprechstunde mit nichtakuten vestibulären Beschwerden im Fokus. Für Anamnese, klinische und apparative Diagnostik bei Patienten mit **akuten vestibulären Syndromen**

Akute vestibuläre Syndrome

(AVS) sei auf die Arbeiten von Newman-Toker et al. [1], Newman-Toker und Edlow [2], Kattah [3], Rosengren et al. [4] und Mantokoudis et al. [5] verwiesen.

## Anamnese

Der Anamnese kommt bei Patienten mit dem Leitsymptom Schwindel eine zentrale Bedeutung zu, der prädiktive diagnostische Wert wird mit bis zu 80 % angegeben [6]. Für viele vestibuläre Syndrome gibt es zudem keinen beweisenden diagnostischen Test [7, 8]. Neben der Informationsgewinnung und dem Erkennen von pathognomonischen Symptomkonstellationen vestibulärer Syndrome [9] ist das Anamnesegespräch auch Voraussetzung für den Aufbau einer vertrauensvollen und tragfähigen Arbeitsbeziehung zwischen Arzt und Patient [10]. Ohne diese wären bei der Patientin in der Kasuistik gar keine weitere Untersuchung und damit keine Diagnosestellung und Therapie möglich. Es lohnt sich also auf jeden Fall, in die Kunst der Anamneseerhebung zu investieren.

Die Arbeiten von Fife [9], Schmäl [11] und Strupp et al. [12] geben eine umfassende aktuelle Übersicht zum Thema der Anamneseerhebung bei Schwindel. Im Folgenden werden einige typische alltägliche Herausforderungen und mögliche Lösungsansätze herausgegriffen.

### Herausforderung 1: Erwartungen und Ziele definieren

Der Patient, der mit dem Aktenordner voller Vorbefunde erwartungsvoll das Sprechzimmer betritt („Sie sind meine letzte Hoffnung.“) hat naturgemäß andere Erwartungen an die folgende Konsultation als der Arzt, der sich mit einer völlig ausgebuchten Schwindelsprechstunde konfrontiert sieht. Daher ist es wichtig, dass beide Seiten zu Beginn ihre Ziele offen kommunizieren und den **Zeitrahmen**

Zeitrahmen

definieren. Vonseiten des Arztes könnte dies so klingen: „Wir haben jetzt 30 min zur Verfügung. Mein Ziel für diese Zeit ist es, dass Sie mir Ihre Beschwerden schildern und ich eine erste klinische Untersuchung durchführe. Abhängig davon planen wir dann einen Termin für einige apparative Zusatzuntersuchungen. Ist das für Sie so in Ordnung?“ An die Antwort des Patienten sollte sich die Frage des Arztes anschließen: „Was erwarten Sie von dem Termin heute?“ Dabei kann auch darauf hingewiesen werden, dass bei größerem Zeitbedarf die Möglichkeit eines Folgetermins besteht. So können unterschiedliche Erwartungen von Arzt und Patient gleich zu Beginn des Gesprächs thematisiert werden und führen nicht zu Missverständnissen und Unzufriedenheit im weiteren Verlauf [9, 13].

#### Merke

Sprechen Sie mit Ihrem Patienten offen über Ziele und Erwartungen in Bezug auf den aktuellen Konsultationstermin.

### Herausforderung 2: „ausschweifende“ Anamnese

Aus Sorge, dass eine ausschweifende Anamnese des Patienten die Konsultationszeit „sprengt“, neigen viele Ärzte dazu, von Beginn an geschlossene Fragen zu stellen und den Patienten bei der Schilderung seiner Beschwerden zu unterbrechen – das erste Mal im Mittel bereits nach 11–24 s [14]. Dabei ist dies gar nicht notwendig: Im Durchschnitt geben die Patienten nach etwa 1,5 min freier Rede die Gesprächsleitung verbal oder nonverbal an den Arzt zurück. Bei rund 80 % der Patienten bleibt die anfängliche freie Redezeit unter 2 min [15]. In dieser Zeit erhalten Sie nicht nur wichtige anamnestische Informationen aus erster Hand, Sie lernen auch den Patienten als Person kennen, insbesondere seine Vorstellungen von Gesundheit und Krankheit (**individuelles Krankheitskonzept**

Individuelles Krankheitskonzept;

[16]). Für den Patienten ist diese Möglichkeit der freien Rede entscheidend für die Vertrauensbildung zum Arzt. Während die gefühlte Gesprächsdauer auf Seiten des Patienten – und damit die Patientenzufriedenheit – zunimmt, kommt es durch die anfängliche freie Redezeit von 2 min in der Regel zu keiner objektiven Zunahme der Gesamtkonsultationszeit [14]. Daher ist es empfehlenswert, die Anamneseerhebung mit einer offenen Frage an den Patienten zu beginnen [9].

#### Merke

„Listen to the patient: He is telling you the diagnosis“ (Sir William Osler) – die ersten 2 min der Anamneseerhebung gehören dem Patienten.

Wenn Sie nach dieser Zeit bemerken, dass der Patient nicht zum Ende kommt und noch wichtige Informationen fehlen, können Sie an diesem Punkt mit gezieltem Nachfragen beginnen. Hier können Gedankenstützen wie SO STONED [17] und DISCOHAT [18] für eine systematische Erfassung der Informationen nützlich sein (Tab. 1 und 2; siehe auch „Herausforderung 3“ und „Herausforderung 4“).

**Table 1:** 

**S**	„Symptom“	Symptomqualität Cave: eingeschränkte Reliabilität und diagnostische Trennschärfe (siehe Text)
**O**	„How often?“	Frequenz
**S**	„Since when?“	Sowohl Zeitpunkt als auch assoziiertes Ereignis, z. B. Kopftrauma
**T**	„Trigger“	Unterscheidung zwischen provozierenden und exazerbierenden Faktoren (siehe Text)
**O**	„Otological symptoms“	Cave: Otologische Begleitsymptome schließen eine zentralvestibuläre Erkrankung wie einen AICA-Infarkt nicht aus [2, 19]
**N**	„Neurological symptoms“	Bei akutem vestibulärem Syndrom: „deadly Ds“ (Diplopie, periorale Dysästhesie, Dysphagie, Dysarthrie) als „red flags“ für einen Hirnstamminfarkt [20]
**E**	„Evolution of symptoms“	Entwicklung im Laufe der Zeit: z. B. gleichbleibend, undulierend, zu-/abnehmend
**D**	„Duration of symptoms“	Dauer von Schwindel und assoziierten Symptomen

*AICA* Arteria inferior anterior cerebelli

**Table 2:** 

**D**	„Darkness“	Zunahme der Beschwerden in Dunkelheit und auf unebenem Boden
**I**	„Imbalance“	Gleichgewichtsstörungen beim Stehen und Gehen
**S**	„Supermarket effect“	Zunahme der Beschwerden bei Blick auf Bewegtes (visuelle Dominanz) und in Menschenansammlungen (Cave: auch Symptom bei PPPD, siehe Text)
**C**	„Cognitive complaints“	Störung von Konzentration, Aufmerksamkeit, Gedächtnis, räumlichem Orientierungsvermögen (Navigation) und „double tasking“
**O**	„Oscillopsia“	Scheinbewegungen der Umgebung bei Kopfbewegung
**H**	„Head movements“	Zunahme der Schwindelbeschwerden bei schneller Kopfbewegung
**A**	„Autonomic complaints“	Dysfunktion des autonomen Nervensystems (selten)
**T**	„Tiredness“	Verstärkte Ermüdbarkeit aufgrund der Schwindelsymptome

*PPPD* „Persistent postural perceptual dizziness“

Die Anamnese gestaltet sich außerdem häufig ausschweifend, wenn der Patient gleich zu Beginn sehr ausführlich von Voruntersuchungen und -befunden sowie den Einschätzungen Dritter zu seinem Krankheitsbild berichtet (siehe „Kasuistik“). Hier kann der Patient folgendermaßen wieder behutsam zur eigentlichen Anamneseerhebung zurückgeführt werden [9]: „Es ist gut, dass Sie die Vorbefunde mitgebracht haben. Wir schauen uns das nach unserem Gespräch an. Für mich ist es jetzt am wichtigsten, wie *Sie* das Ganze erleben.“ Am Ende des Anamnesegesprächs sieht sich der Arzt oft mit einer Fülle an belastenden Symptomen für den Patienten konfrontiert. Um hier einen Eindruck zu bekommen, welches der Probleme am vordringlichsten für den Patienten ist, empfiehlt sich die Frage: „Wenn ich heute eines Ihrer gesundheitlichen Probleme lösen könnte, welches sollte es sein?“

### Herausforderung 3: Beschreibung des Symptoms Schwindel

Die Schwierigkeit, das Symptom Schwindel in Worte zu fassen, beginnt schon bei der Beschreibung der Symptomqualität (z. B. Drehen, Schwanken, Liftgefühl etc.). Hier zeigte eine Untersuchung von Newman-Toker et al., dass etwa die Hälfte der Patienten in 2 Konsultationen eine unterschiedliche führende Symptomqualität angab [21]. Zur schlechten Reliabilität dieses Faktors kommt noch eine geringe Trennschärfe bei der Unterscheidung einzelner vestibulärer Syndrome hinzu [22].

#### Merke

Vestibuläre Diagnosen sollten nie allein auf der Basis der Symptomqualität gestellt oder ausgeschlossen werden.

Der zeitliche Verlauf („timing“) und auslösende Faktoren („triggers“) von Schwindelbeschwerden zeigen sowohl eine höhere Reliabilität als auch eine höhere Trennschärfe. Daher sollten diese beiden Faktoren immer erfragt werden, bevor sich eine gezielte neurootologische Untersuchung mit ggf. apparativer Diagnostik anschließt (TiTrATE = „timing, triggers and targeted examination“; [2]).

Beim **„timing“**

„Timing“

wird insbesondere zwischen akuten, episodischen und chronischen vestibulären Syndromen unterschieden (siehe [2, 23] zur Zuordnung einzelner Krankheitsbilder). Bei den **Triggern**

Trigger

muss zwischen provozierenden und exazerbierenden Faktoren differenziert werden [9, 20]. Berichtet ein Patient beispielsweise über Schwindel bei Kopfbewegungen, sollte nachgefragt werden, ob dies zu einem neu aufgetretenen Schwindel aus völliger Symptomfreiheit führt (**Provokation**

Provokation)

oder ob ein bereits vorhandenes Schwindelgefühl durch Kopfbewegungen verstärkt wird (**Exazerbation**

Exazerbation).

Ersteres würde eher für einen **benignen paroxysmalen Lagerungsschwindel (BPLS)**

Benigner paroxysmaler Lagerungsschwindel (BPLS)

sprechen, Letzteres für eine **bilaterale Vestibulopathie**

Bilaterale Vestibulopathie

oder eine inkomplett kompensierte einseitige **chronische vestibuläre Unterfunktion**

Chronische vestibuläre Unterfunktion.

Um weitere Dimensionen vestibulärer Syndrome jenseits von „timing“ und „triggers“ systematisch im Anamnesegespräch zu erfassen, kamen in den letzten Jahren verschiedene Merkhilfen (Akronyme) wie SO STONED (Tab. 1) und DISCOHAT (Tab. 2) hinzu (Zeitbedarf der Erhebung jeweils etwa 2 min). Letztere wurde spezifisch für die Erfassung chronischer uni- und bilateraler Vestibulopathien entwickelt (siehe auch „Herausforderung 4“).

#### Merke

Gedächtnisstützen wie TiTrATE, SO STONED und DISCOHAT helfen bei der systematischen Erfassung und Einordung von vestibulären Syndromen.

Am Ende der Anamneseerhebung sollte der Arzt die vom Patienten geschilderten Beschwerden nochmals kurz in eigenen Worten zusammenfassen. Am Schluss sollte immer die Nachfrage stehen: „Habe ich das richtig verstanden? Haben wir noch etwas Wichtiges vergessen, was Sie mir über Ihren Schwindel berichten wollten?“ Damit stellen Sie sicher, dass Sie alle für den Patienten relevanten Informationen erfasst und richtig verstanden haben [9]. Durch das **aktive Zuhören**

Aktives Zuhören

signalisieren Sie Ihrem Patienten Wertschätzung, was wiederum die vertrauensvolle Arzt-Patient-Beziehung stärkt [10, 13]. Und schließlich strukturieren Sie so die Konsultationszeit und können zum nächsten Schritt, der klinisch-neurootologischen Untersuchung, übergehen.

### Herausforderung 4: ein Patient – mehrere Schwindelentitäten

Viele Patienten, gerade an spezialisierten Schwindelzentren, leiden unter mehr als einer Schwindelentität, die Angaben in der Literatur variieren zwischen 16 und 50 % [9, 20]. Zudem ist bei etwa der Hälfte der Patienten mit chronischen bzw. chronisch-rezidivierenden Schwindelbeschwerden eine zusätzliche funktionelle oder psychische **Komorbidität**

Komorbidität

zu erwarten [20]. Eine Zusammenfassung der häufigsten Kombinationen findet sich bei Fife [9].

Hier empfiehlt sich ein 4‑stufiges Vorgehen, um die einzelnen Schwindelentitäten zu identifizieren [20]:

1. Zunächst wird nach dem Vorhandensein von Schwindelattacken gefragt, welche mittels SO STONED näher charakterisiert werden. Beschreibt der Patient mehrere Arten von Attackenschwindel, z. B. typisch für M. Menière und BPLS, wird dieses Prinzip für jede der Entitäten angewandt.
2. Danach folgt die Frage: „Haben Sie zwischen den Attacken auch Probleme mit dem Gleichgewicht?“ Wird diese bejaht, wird eine mögliche zugrunde liegende chronische vestibuläre Störung nach dem DISCOHAT-Akronym exploriert.
3. Im dritten Schritt wird ein Screening nach möglichen funktionellen oder psychischen Erkrankungen durchgeführt. In Tab. 3 sind typische anamnestische und klinische Hinweise für funktionelle Schwindelsyndrome, z. B. **PPPD („persistent postural perceptual dizziness“)**
  PPPD („persistent postural perceptual dizziness“)
  zusammengefasst (siehe [24, 25, 26] für weitere Informationen zu Pathophysiologie, Diagnosestellung und Therapie).
4. Schließlich erfolgt im 4. Schritt die Synthese der vorangegangenen Schritte mit der Stellung einer oder mehrerer Diagnosen.

**Table 3:** 

*Anamnese*
– Dauer über Monate – Besserung der Symptome bei Ablenkung und bei Genuss geringer Alkoholmengen – Starke situative Abhängigkeit der Beschwerden – Visuelle Dominanz (z. B. „supermarket effect“; siehe Tab. 2) – Diskrepanz: große Sturzangst – bisher keine Stürze – Ausgeprägtes Vermeidungsverhalten – Aktuelle Symptome oder Vorgeschichte für Angststörungen und/oder Depression
*Klinische Untersuchung*
– Abnahme des Schwankens bei zunehmendem Schwierigkeitsgrad in den Stand- und Gangprüfungen – Deutliche Verbesserung der Gangunsicherheit bei minimaler Unterstützung – Deutliche Diskrepanz zwischen subjektivem Symptom und objektivem Befund

Mit etwas Übung lassen sich so auch bei komplexen Patienten an einem tertiären Zentrum verschiedene Schwindelentitäten innerhalb von 15 min erfassen [20]. Bei der Patientin aus der Kasuistik kristallisierten sich auf diese Weise 3 verschiedene Schwindelentitäten heraus:

1. Rechtsdrall für Sekunden beim Hinlegen auf den Rücken,
2. kurzer Schwankschwindel beim Vorbeugen und Reklinieren des Kopfes,
3. Dauerbenommenheit und Gangunsicherheit.

Vonseiten der Anamnese sprachen folgende Faktoren zumindest für eine funktionelle Schwindelkomponente (Tab. 3):

- Dauer über Monate,
- situative Trigger,
- ausgeprägtes Vermeidungsverhalten und daraus resultierende soziale Isolation,
- ausgeprägte Sturzangst und Kinesiophobie bisher ohne Stürze.

Mögliche zugrunde liegende strukturelle vestibuläre Erkrankungen wurden in der folgenden klinischen und apparativen Untersuchung weiter abgeklärt (siehe „Klinisch-neurootologische Untersuchung“ und Folgeartikel).

### Herausforderung 5: Diskrepanz zwischen Symptom und Befund

Häufig sieht sich der Neurootologe mit der Situation konfrontiert, dass die subjektive Symptomlast des Patienten nicht mit den objektiven Befunden in der klinisch-neurootologischen Untersuchung und in den apparativen Tests übereinstimmt. Dies ist bis zu einem gewissen Grad normal: Mehrere Studien haben ergeben, dass vestibuläre Untersuchungsbefunde und die Symptomschwere bzw. die Lebensqualität nicht direkt miteinander korrelieren [20, 28]. Dies trifft insbesondere für episodische vestibuläre Symptome wie den M. Menière zu, wo sich im attackenfreien Intervall häufig keine pathologischen vestibulären Befunde zeigen, die Lebensqualität von den Patienten aber dennoch als stark eingeschränkt empfunden wird [29].

Bei einer außergewöhnlich großen Inkongruenz zwischen Symptomen und Befunden sollte in Abhängigkeit vom Kontext auch an eine funktionelle Krankheitskomponente gedacht werden. Wenn sich beispielsweise bei einem Patienten nach einer **Neuritis vestibularis**

Neuritis vestibularis

im Laufe der Zeit in den Untersuchungsbefunden eine zunehmende zentrale Kompensation zeigt, der Patient aber immer noch nicht in der Lage ist, seinen Alltag zu bewältigen, ist dies nicht mehr allein durch die Neuritis zu erklären, sondern es sollten auch andere Faktoren, wie eine sekundäre PPPD, erwogen werden [20].

### Herausforderung 6: kognitive Verzerrungen

Kognitive Verzerrungen („bias“) sind sowohl aufseiten des Patienten als auch des Arztes vorhanden. Insbesondere besteht auf beiden Seiten die Neigung, alle Symptome des Patienten auf *eine* gemeinsame Ursache zurückzuführen [9, 20]. So war die Patientin aus der Kasuistik davon überzeugt, dass der Schwindel und die Gangunsicherheit allein durch die HWS-Beschwerden bedingt seien, da der Schwindel bei jeder Kopfbewegung zunähme und die Schmerzen im Bereich des Atlas erstmals nach der Reposition des Lagerungsschwindels aufgetreten seien.

Um derartige kognitive Verzerrungen aufzudecken, empfiehlt es sich, den Patienten nach seiner **individuellen Krankheitstheorie**

Individuelle Krankheitstheorie

[16] zu befragen: „Was glauben *Sie*, woher die Beschwerden kommen?“ Die Antwort auf diese Frage ermöglicht Ihnen einen Einblick in das Verständnis des Patienten von Gesundheit und Krankheit, und Sie können seine individuellen Krankheitskonzepte bei der anschließenden Erläuterung der Diagnose und der Beratung über die Therapie wieder aufgreifen (siehe Folgeartikel). Ohne diese Einsicht arbeiten Sie während der gesamten Konsultation gegen einen unsichtbaren Widerstand an, falls eine Diskrepanz zwischen Ihrem Krankheitskonzept und dem des Patienten besteht [9].

An dieser Stelle ist zu beachten, dass auch Ärzte und Therapeuten kognitiven Verzerrungen unterliegen. Gerade bei Patienten mit einer bekannten psychiatrischen Grunderkrankung sollte der Neurootologe nicht der Versuchung erliegen, alle Schwindelsymptome „auf die Psyche zu schieben“, auch wenn diese zuerst etwas bizarr klingen mögen, wie z. B. bei **Syndromen des dritten Fensters**

Syndrome des dritten Fensters

[23].

### Herausforderung 7: Umgang mit Emotionen

Die emotionale Dimension von Schwindel ist oft der sprichwörtliche „elephant in the room“: Obwohl klar und deutlich vorhanden, vermeiden es sowohl der Arzt als auch der Patient, darüber zu sprechen. Negative Emotionen wie Angst, Verzweiflung, Wut oder Frustration sind häufig schambesetzt. Patienten befürchten, beim Zulassen dieser Gefühle vom Arzt in die „Psychoecke“ gedrängt zu werden. Vonseiten des somatisch ausgebildeten Arztes besteht oft eine gewisse Unsicherheit im Umgang mit Emotionen. Hinzu kommt wieder die Sorge vor einem Überschreiten der vorgesehenen Konsultationszeit. Ich möchte Sie trotzdem dazu ermutigen, die Emotionen des Patienten in der Anamneseerhebung aufzugreifen.

Tun wir dies nicht, stehen diese Emotionen zwischen uns und dem Patienten und blockieren den Aufbau eines vertrauensvollen Arbeitsverhältnisses. Im Extremfall entladen sich die Emotionen spontan und unvorhergesehen wie der sprichwörtliche „Dampfkochtopf“. Beides „kostet“ am Ende mehr Zeit als ein professionell-empathischer Umgang mit Emotionen. Hier hat sich z. B. das **NURSE(„naming“/„understanding“/„respecting“/„supporting“/„exploring“)-Prinzip**

NURSE-Prinzip

[10] bewährt (Tab. 4).

**Table 4:** 

**N**	„Naming“	Emotionen benennen und ansprechen
**U**	„Understanding“	Emotionen verstehen und Verständnis vermitteln
**R**	„Respecting“	Bemühungen des Patienten wertschätzen, die Situation zu bewältigen
**S**	„Supporting“	Unterstützung anbieten
**E**	„Exploring“	Ursachen für die Emotionen ergründen

Bei der Patientin aus der Kasuistik war es ein entscheidender Schritt, dass die Emotionen durch die behandelnde Ärztin benannt („Für mich hört es sich so an, als hätten Sie große Angst davor, Ihren Kopf zu bewegen.“) und weiter exploriert wurden („Warum ist das so?“). Die Patientin fühlte sich ernst genommen und teilte daraufhin ihre große Sorge mit, dass durch jegliche Bewegung des Kopfes der Schwindel und die Schmerzen in der HWS zunehmen könnten. Sie berichtete dabei von einer negativen Erfahrung, wo bei einem Arztbesuch genau dies eingetreten sei. Diese Information ermöglichte es der Ärztin, die folgenden Untersuchungsschritte unter diesem Aspekt mit der Patientin zu besprechen und ihr die Angst vor Symptomverschlimmerung zumindest teilweise zu nehmen. Ohne die Thematisierung der Emotion Angst wäre eine klinische Untersuchung wegen mangelnder Kooperation der Patientin kaum möglich gewesen.

## Klinisch-neurootologische Untersuchung

Die klinisch-neurootologische Untersuchung im Anschluss an die Anamneseerhebung ist der erste Schritt der **„targeted examination“**

„Targeted examination“

aus dem TiTrATE-Algorithmus. Für Übersichtsartikel zu diesem Thema sei auf die Arbeiten von Strupp et al. [12], Straumann [30] sowie Brune und Eggenberger [31] verwiesen. Details zur klinischen **Nystagmusprüfung**

Nystagmusprüfung

finden sich bei Strupp et al. [32] und Müller [33], die **Gangprüfung**

Gangprüfung

wird sehr anschaulich von Jahn et al. [27] beschrieben. Videos zu Untersuchungstechniken und typischen Befunden sind online frei zugänglich in der Neuro-Ophthalmology Virtual Education Library (NOVEL; [34]) verfügbar.

Der folgende Abschnitt widmet sich typischen Herausforderungen und Fallstricken, welche insbesondere bei Patienten mit HWS-Problemen, mit Okulomotorikstörungen oder bei ängstlichen Personen angetroffen werden. Charakteristische Untersuchungsbefunde und „red flags“ einzelner vestibulärer Syndrome werden in einem der Folgeartikel weiter vertieft.

### Merke

Die neurootologische Untersuchung beginnt bereits mit dem Gang ins Untersuchungszimmer.

Wann immer es organisatorisch und räumlich möglich ist, sollte der behandelnde Arzt bzw. die Ärztin den Patienten persönlich aus der Wartezone abholen und mit ihm gemeinsam zum Untersuchungszimmer gehen. Dabei zeigt sich bereits, wie sicher der Patient auf den Beinen ist und ob er eine Gehhilfe benötigt [9]. Bei der Patientin aus der Kasuistik präsentierte sich hier ein extrem unsicheres, ängstliches Gangmuster („walking on ice“), welches sich bereits bei minimaler Unterstützung (Festhalten am Arm der Mutter) und bei **„dual tasking“**

„Dual tasking“

(Unterhaltung mit der Ärztin) deutlich besserte. Diese Beobachtungen sprechen für eine funktionelle Komponente der Gangstörung ([27]; Tab. 3), wobei andere mögliche Ursachen bei der folgenden Untersuchung weiter abgeklärt wurden.

### Reihenfolge der einzelnen Untersuchungen

Prinzipiell ist es sinnvoll, die einzelnen Untersuchungsschritte nach einem festen Muster durchzuführen, um keine Tests zu vergessen. In individuellen Fällen kann die Reihenfolge abhängig von der Gesamtkonstellation angepasst werden. Dabei sollte bedacht werden, dass Untersuchungen, welche mit großer Wahrscheinlichkeit beim Patienten Schwindel auslösen, am Ende des Untersuchungsganges erfolgen sollten. Ein Patient mit BPLS kann beispielsweise nach den **Lagerungsmanövern**

Lagerungsmanöver

Schwindel und Übelkeit so stark verspüren, dass hierdurch die folgenden Untersuchungen verfälscht werden oder im Extremfall gar nicht mehr möglich sind. Daher sollten diejenigen Tests, für die der Patient seine bestmögliche Balance benötigt (z. B. Stand- und Gangprüfung), auch gleich zu Beginn stattfinden.

Aus der Erfahrung der Autorin hat sich im Normalfall das folgende Vorgehen bewährt.

#### Im Stehen:

1. Stand- und Gangprüfung: Romberg-Versuch auf festem Boden (ggf. auch im Tandemstand) und auf Schaumstoff („Romberg on foam“ [„FRomberg“]); Unterberger-Tretversuch; Gehen in unterschiedlichen Tempi (selbst gewählt, langsam, schnell), ggf. auch im Seiltänzergang oder rückwärts; Durchführung mit „dual tasking“ bei V. a. funktionelle Störungen

#### Im Sitzen auf der Liege:

2. Armvorhalteversuch, zerebelläre Koordination (Diadochokinese: Finger-Nase-Versuch, Knie-Hacke-Versuch), ggf. Prüfung der bimalleolären Pallästhesie
3. Untersuchung der Okulomotorik: Lichtreaktion der Pupillen, wechselseitiger Abdeckversuch, glatte Blickfolge (inkl. Beurteilung Nystagmus unter Fixation, Blickrichtungsnystagmus), Sakkaden, orientierende Prüfung des Gesichtsfeldes
4. Kopfimpulstest („head impulse test“, HIT)
5. Ggf. Zusatzuntersuchungen der Okulomotorik: Fixationssuppression des vestibulookulären Reflexes (VOR), visuell verstärkter VOR (vVOR)
6. Optokinetischer Nystagmus
7. Nystagmusanalyse mit Frenzel- oder Videobrille: Spontan- und Kopfschüttelnystagmus
8. Lagerungsmanöver: Dix-Hallpike-Manöver für die vertikalen, Supine-roll- oder Pagnini-McClure-Manöver für die horizontalen Bogengänge

#### Erläuterungen:

Zu 1: Der **FRomberg-Test**

FRomberg-Test

ist ein sensitiver Indikator für eine uni- oder bilaterale Vestibulopathie [30]. Bei der Untersuchung der vestibulospinalen Reaktionen im Dual-task-Modus zeigen Patienten mit einer ängstlichen oder funktionellen Störung häufig eine deutliche Verbesserung der posturalen Kontrolle ([24, 27]; Details siehe Folgeartikel).

Zu 2: Diese Untersuchungen lösen in der Regel keinen Schwindel beim Patienten aus und eignen sich daher besonders gut für den Untersuchungsbeginn. Die Untersuchung der **Pallästhesie**

Pallästhesie

(Vibrationsempfinden) mit der Rydel-Seiffer-Stimmgabel (64–128 Hz) sollte auch vom HNO-Arzt bei Patienten mit Stand- und Gangunsicherheit als Screeningtest auf eine **distal-sensible Polyneuropathie**

Distal-sensible Polyneuropathie

durchgeführt werden (Normwert bimalleolär: 4–5,5/8, abhängig vom Alter [35]).

Zu 3: Bei Patienten mit visueller Dominanz (z. B. bei vestibulärer Migräne oder PPPD) kann bereits das Fixieren des sich bewegenden Zielpunkts bei der Prüfung der glatten Blickfolge und Sakkaden Schwindel auslösen; dies ist ein wichtiger diagnostischer Hinweis. Die Prüfung der Okulomotorik sollte immer vor dem Kopfimpulstest erfolgen, um sicherzustellen, dass der efferente Schenkel des VOR (d. h. Augenmuskeln und deren Innervation) intakt ist ([30]; Details siehe „Patienten mit Okulomotorikstörungen“).

Zu 5: Die Beurteilung der Fixationssuppression des VOR (typisch bei zerebellären Läsionen des Flokkulus/Paraflokkulus; [32]) und des visuell verstärkten VOR (sakkadiert bei einer Kombination aus bilateraler Vestibulopathie und zerebellärem Syndrom; [36]) ist nur in Zusammenschau mit dem **Kopfimpulstest**

Kopfimpulstest

(Prüfung des VOR) möglich. Daher sollten diese Spezialuntersuchungen bei Bedarf nach dem Kopfimpulstest erfolgen.

Zu 6: Die Untersuchung mit der **optokinetischen Trommel**

Optokinetische Trommel

ist nicht nur ein effektiver Screeningtest zur gleichzeitigen Untersuchung der glatten Blickfolge und der Sakkaden, sondern dient auch der Identifikation von Patienten mit visueller Dominanz ([30]; Details siehe Folgeartikel).

#### Merke

Führen Sie „schwindelerregende“ Tests am Ende des Untersuchungsgangs durch.

### Dokumentation der Befunde

Es empfiehlt sich, die Augenbewegungen der klinisch-neurootologischen Untersuchung mit einem **tragbaren Videookulographiesystem**

Tragbares Videookulographiesystem,

z. B. einer Video-HIT-Kamera, aufzuzeichnen. Die Untersuchungszeit verlängert sich im Vergleich zur **Frenzel-Brille**

Frenzel-Brille

nicht wesentlich, und während der Untersuchung können die Augenbewegungen vergrößert auf dem Monitor beobachtet werden. Zudem ermöglicht diese objektive Dokumentation eine Nachbefundung bzw. Offlineanalyse komplexer Untersuchungsbefunde und eine telemedizinische Mitbeurteilung durch andere Kollegen [4, 5].

Wenn sich bei episodischen vestibulären Syndromen während der Untersuchung im freien Intervall kein Nystagmus nachweisen lässt, sollte dem Patienten empfohlen werden, bei der nächsten Schwindelattacke – wenn möglich – seine Augenbewegungen mit der Smartphonekamera im Sinne eines vestibulären „event monitoring“ zu filmen [7, 37, 38].

### Patienten mit orthopädischen Problemen, insbesondere an der HWS

Wenn der Romberg-Test wegen orthopädischer Probleme nicht im Stehen durchführbar ist, kann auch das Schwanken des Oberkörpers im Sitzen bei geschlossenen Augen, z. B. im Rahmen des Armvorhalteversuchs, beurteilt werden. Bei V. a. eine chronische vestibuläre Unterfunktion empfiehlt es sich, den Patienten bzw. die Patientin auf einem Kissen sitzen zu lassen, um die somatosensorischen Afferenzen zu reduzieren und so die vestibuläre Störung zu demaskieren. Wie bei den Untersuchungen im Stehen kann auch in dieser Situation der Effekt von „dual tasking“ getestet werden.

Bei Problemen mit der HWS empfiehlt sich zunächst eine Evaluation der Beschwerden zusammen mit dem Patienten (z. B.: Welche Bewegungen sind von orthopädischer Seite überhaupt erlaubt? Welche Bewegungseinschränkung besteht in welche Richtungen? Auslösung von Schwindel durch bestimmte Bewegungen? Bisherige Therapien?). Danach wird der Patient gebeten zu zeigen, wie weit er den Kopf in welche Richtungen ohne Auslösung von Symptomen wie Schwindel oder Schmerzen bewegen kann. Nach Erfahrung der Autorin ist es sinnvoll, anschließend mit dem Patienten zu besprechen, welche klinischen Untersuchungen vorgesehen sind und wie weit der Kopf dabei bewegt werden muss. Generell ist es besser, den Patienten die Kopfbewegungen selbst durchführen zu lassen, als auf eine Untersuchung komplett zu verzichten, z. B. bei Prüfung des Kopfschüttelnystagmus. Durch diese Maßnahmen bleibt die Selbstwirksamkeit des Patienten erhalten, was zu einer Stärkung des Vertrauensverhältnisses zwischen Arzt und Patient und damit zu einer besseren Kooperation während der Untersuchung führt [10].

Durch dieses Vorgehen ließ auch die Patientin aus der Kasuistik Kopfbewegungen bei der klinisch-neurootologischen Untersuchung in einem gewissen Masse zu. Für die Prüfung des Kopfimpulstests zeigte sie, wie weit sie den Kopf zur Seite drehen konnte (> 45° nach rechts und links). Danach wurde sie gebeten, den Kopf um 10–20° selbst zu bewegen, was für sie gut möglich war. Nach dieser Vorbereitung war eine Durchführung des Kopfimpulstests durch die Ärztin möglich, wobei der Kopf in kleinen Amplituden zunächst langsam und dann schneller bewegt wurde. Durch eine Unterhaltung wurde die Patientin von der eigentlichen Untersuchung abgelenkt, sodass sie schließlich überrascht war, dass diese vorbei war, bevor sie etwas davon bemerkt hatte. Der VOR zeigte sich auf beiden Seiten normal, womit ein höhergradiges peripher-vestibuläres Defizit ausgeschlossen werden konnte. Ein analoges stufenweises Vorgehen für den Kopfimpulstest empfiehlt sich auch bei ängstlichen Patienten (siehe unten).

### Patienten mit Okulomotorikstörungen

Es ist essenziell, den Patienten nach bekannten Augenbewegungsstörungen inklusive **Schielens**

Schielen

zu fragen und den Abdecktest sowie die Prüfung von glatter Blickfolge und Sakkaden vor dem Kopfimpulstest durchzuführen (siehe oben). Eine zuverlässige Interpretation des Kopfimpulstests ist nur möglich, wenn der efferente Schenkel des VOR (d. h. die Augenmuskeln und ihre Innervation) intakt ist. Gerade beim Schielen können die Ergebnisse des Kopfimpulstests verfälscht werden, insbesondere wenn das dominante Auge abhängig von der Blickrichtung wechselt. Hier empfiehlt es sich, das schielende Auge für die Untersuchung abzukleben. Gleiches gilt für periphere Augenmuskelparesen [30, 31].

#### Merke

Prüfen Sie die Okulomotorik vor der Durchführung des Kopfimpulstests.

### Ängstliche Patienten

Gerade bei der Gruppe der ängstlichen Patienten ist es unverzichtbar, bereits im Anamnesegespräch ein vertrauensvolles Arzt-Patient-Verhältnis aufzubauen. In diesem Rahmen sollten Ängste bezüglich der anstehenden Untersuchung thematisiert und nach dem NURSE-Schema bearbeitet werden [10, 20]. Des Weiteren ist es wichtig, dem Patienten Informationen über die anstehenden Untersuchungen zu vermitteln und die zentrale Bedeutung seiner Mitarbeit positiv hervorzuheben. Auf diese Weise können Arzt und Patient im Sinne einer partizipativen Entscheidungsfindung vereinbaren, welche Untersuchungen durchgeführt werden und auf welche eventuell verzichtet werden kann [39]. Angst bedeutet häufig auch Angst vor Kontrollverlust. Daher ist es empfehlenswert, den Patienten möglichst viele Bewegungen selbst durchführen zu lassen, um seine Selbstwirksamkeit zu stärken. Auch sollte die Möglichkeit von Pausen angeboten werden. Dieses Vorgehen mag dem klinisch tätigen HNO-Arzt zunächst etwas umständlich erscheinen, kann aber entscheidend dafür sein, dass wir am Ende die notwendigen diagnostischen Informationen erhalten, um unseren Patienten effektiv zu helfen.

Besonders wichtig ist dieses behutsame Vorgehen bei Patienten mit BPLS. Hier ist es für die erfolgreiche Durchführung der diagnostischen und therapeutischen Manöver essenziell, dass der schwindel- und häufig auch angstgeplagte Patient versteht, warum eine erfolgreiche Therapie nur über eine temporäre Verstärkung der Schwindelbeschwerden möglich ist. Für die Erläuterung der Pathophysiologie sowie der diagnostischen und therapeutischen Schritte bieten sich animierte Modelle an, die der Arzt auf seinem Smartphone in der Kitteltasche immer bei sich hat [40]. Bei extrem ängstlichen Patienten mit BPLS ist auch die Gabe eines Anxiolytikums (z. B. Lorazepam) vor der Untersuchung sowie das Hinzuziehen einer Hilfsperson während der Lagerungen zu erwägen. Die Anwendung von speziellen Behandlungsstühlen zur Diagnose und Therapie des BPLS [41] wird im Folgeartikel zur apparativen Vestibularisdiagnostik näher erläutert.

Da bei der Patientin aus der Kasuistik in der Vorgeschichte ein BPLS bekannt war, wurden hier auch Lagerungsmanöver durchgeführt. Nach dem Aufbau eines vertrauensvollen Verhältnisses ließen wir die Patientin zunächst selbst diejenigen Positionsänderungen vornehmen, welche in der Vergangenheit Schwindel induziert hatten. Als dabei kein Schwindel auftrat, war die Patientin auch zur Durchführung der „klassischen“ Lagerungsmanöver durch die behandelnde Ärztin bereit. Dabei zeigte sich kein BPLS-typischer Nystagmus, und es konnte auch kein Schwindel ausgelöst werden. Diese Erfahrung führte bei der Patientin zu der Frage: „Dann ist das jetzt gar kein Lagerungsschwindel?“, welche von ärztlicher Seite als Einstieg in das anschließende aufklärende Gespräch genutzt wurde – mehr dazu in den Folgeartikeln.

## Fazit für die Praxis

- Keine Angst vor „schwierigen“ Schwindelpatienten!
- Nutzen Sie Gedächtnisstützen wie TiTrATE, SO STONED und DISCOHAT für eine umfassende und strukturierte Anamneseerhebung.
- Investieren Sie von Beginn der Konsultation an in eine vertrauensvolle Arbeitsbeziehung zwischen Ihnen und Ihrem Patienten.
- Versuchen Sie, „schwierige Untersuchungsbedingungen“ nicht als Störfaktor, sondern als wichtige diagnostische Information und als Möglichkeit zum Ausbau des Vertrauensverhältnisses zwischen Arzt und Patient zu sehen.
- Haben Sie Geduld – mit Ihren Patienten und mit sich selbst.

## CME-Fragebogen

Welche Information aus der Anamnese besitzt die *geringste* diagnostische Trennschärfe in Bezug auf vestibuläre Syndrome?

Zeitliche Dimension der Beschwerden (akut, episodisch, chronisch)

Begleitsymptome einer Schwindelattacke

Trigger von Schwindelbeschwerden

Dauer einer Schwindelattacke

Symptomqualität

Welche Dimension chronischer Schwindelbeschwerden wird im Akronym DISCOHAT erfasst?

Dauer

Trigger

Oszillopsien

Ohrsymptome

Schweregrad

Wofür steht das „E“ in SO STONED?

Emotionale Auswirkungen des Schwindels

Entwicklung der Schwindelsymptome über die Zeit

Episodischer Schwindel

Erstereignis

Effekt des Schwindels auf die Lebensqualität

Ein 35-jähriger Patient stellt sich in Ihrer Sprechstunde mit einem chronischen Schwankschwindel seit 4 Monaten vor. Welcher Hinweis spricht am ehesten für eine funktionelle Schwindelerkrankung?

Zunahme des Schwankens im Romberg-Test auf unebenem Untergrund

Erlittene Stürze

Abnahme der Beschwerden in belebten Umgebungen

Zunahme der Beschwerden nach dem Genuss geringer Mengen Alkohol

Zunahme der Gangsicherheit beim „dual tasking“

Wofür steht das „S“ im NURSE-Schema zum Erfassen von Emotionen des Patienten?

„Severity“

„Support“

„Since when?“

„Significance“

„Sensitivity“

Wie lange antworten Patienten im Durchschnitt auf eine offene Frage zu Beginn der Anamneseerhebung?

1 min

1,5 min

2,5 min

4 min

6 min

Welche Aussage zur Anamneseerhebung beim Leitsymptom Schwindel trifft am ehesten zu?

Durch die Fortschritte in der apparativen vestibulären Diagnostik hat der Stellenwert der Anamnese in den letzten Jahren abgenommen.

Die Symptomqualität erlaubt eine zuverlässige Zuordnung zu peripher- oder zentral-vestibulären Syndromen.

Die Verwendung von möglichst vielen geschlossenen Fragen führt zu einer effektiven Konsultation.

Zeitlicher Verlauf („timing“) und auslösende Faktoren („triggers“) werden von Patienten zuverlässiger wiedergegeben als die Symptomqualität.

Emotionen des Patienten sollten bei der Erstkonsultation möglichst nicht angesprochen werden.

Ein 20-jähriger Patient mit starkem Auswärtsschielen auf dem rechten Auge kommt in Ihre Sprechstunde. Welche Aussage trifft am ehesten für die neurootologische Untersuchung zu?

Es müssen keine besonderen Maßnahmen getroffen werden.

Bei der Untersuchung der Okulomotorik und des vestibulookulären Reflexes wird das rechte Auge abgeklebt.

Hier muss eine Videookulographie erfolgen, da die klinische Untersuchung der Okulomotorik nicht aussagekräftig ist.

Zunächst sollte ein augenärztliches Konsil erfolgen.

Der Kopfimpulstest kann hier nicht konklusiv beurteilt werden.

Für welche Ursache einer Stand- und Gangunsicherheit ist der FRomberg(„Romberg on foam“)-Test besonders sensitiv?

Vestibulär

Propriozeptiv

Visuell

Kortikal

Orthostatisch

Was testet die Untersuchung mit der optokinetischen Trommel?

Vestibulookulärer Reflex

Sakkaden und glatte Blickfolge

Konvergenz

Gesichtsfeld

Blickhaltefunktion
